# Prevalence and Risk of Dental Erosion Linked to Carbonated Drinks in Adolescents: A Systematic Review and Meta-Analysis

**DOI:** 10.3290/j.ohpd.c_2619

**Published:** 2026-05-11

**Authors:** María Lilia Adriana Juárez López, Maria Fernanda Gutierrez-Moreno, Lizett Castrejón-Delgado, Martha Asunción Sánchez-Rodríguez

**Affiliations:** a María Lilia Adriana Juárez López Dentist, FES Zaragoza, Universidad Nacional Autónoma de México, Mexico. Contributed to the conceptualisation of the study, methodology and data curation. Screened relevant publications, evaluated the possible risk of bias, and wrote the manuscript.; b Maria Fernanda Gutierrez-Moreno Dentist, FES Zaragoza, Universidad Nacional Autónoma de México, Mexico. Participated in the methodology, screened relevant publications, and evaluated the possible risk of bias; c Lizett Castrejón-Delgado Pharm Chem., FES Zaragoza, Universidad Nacional Autónoma de México, Mexico. Contributed in methodology, performed the statistical evaluation. Resolved discrepancies in the risk of bias analysis.; d Martha Asunción Sánchez-Rodríguez Pharm Chem., FES Zaragoza, Universidad Nacional Autónoma de México, Mexico. Contributed to methodology, review, and analysis. Proofread the manuscript, the statistical evaluation, and contributed to the discussion.

**Keywords:** adolescents, carbonated beverages, dental erosion, risk factor

## Abstract

**Purpose:**

To determine the prevalence of dental erosion due to carbonated beverage consumption among adolescents and the association between both variables through a systematic review and meta-analysis.

**Methods and Materials:**

This study was conducted in accordance with the preferred reporting items for systematic review and meta-analysis (PRISMA) guidelines for systematic reviews. A comprehensive literature search was performed in PubMed, Scopus, Web of Science, LILACS, and SciELO. Observational studies were included, and the PEO framework was used to identify dental erosion and non-erosion (outcome) and intake of carbonated beverages (exposure) in adolescents (population). The prevalence of dental erosion and odds ratio (OR) with 95% confidence interval (95%CI) to determine the risk of carbonated beverages were estimated with MedCalc V.23.0.2 and Review Manager (RevMan V.5.4.1) software, respectively. A *P* value < 0.05 was considered to indicate statistical significance.

**Results:**

A total of 24 studies met the eligibility criteria. The pooled sample included 21,541 adolescents, and the overall erosion prevalence was 37.6% (95%CI 26.3, 49.7%). The meta-analysis revealed a significant association between carbonated beverage consumption and dental erosion (OR = 1.98, 95%CI: 1.42–2.77; 7785 participants; I^2^ = 80%; *P* < 0.0001).

**Conclusion:**

Dental erosion is a prevalent condition among adolescents, with a pooled prevalence of 37.6%. Carbonated beverage consumption among adolescents is associated with a 98% increased risk of dental erosion.

Dental erosion is defined as the irreversible loss of tooth structure caused by the chemical action of acids and/or chelating agents of nonbacterial origin. Structural loss due to erosion is a progressive, localised, asymptomatic, and irreversible process. The acids responsible for this process do not originate from intraoral bacterial activity but rather from extrinsic sources such as diet and the environment or from intrinsic sources such as gastric acid.^50^ Among the extrinsic factors, the consumption of beverages with low pH is particularly relevant, as it initially affects the enamel and can reach the dentin and pulp in more advanced stages, leading to hypersensitivity, severe pain, discomfort during mastication, fractures, and even tooth loss.^14,52^

The increasing consumption of soft drinks and other carbonated beverages is a growing trend among adolescents, largely because of changes in dietary habits and lifestyles. The erosive potential of these drinks is linked to both their added sugars and acids and to the presence of carbon dioxide, which produces carbonic acid, and other factors that contribute to their erosive potential are pH, titratable acidity, and their calcium and phosphate concentration. The method, frequency of intake and duration of exposure in the oral cavity are important. There is a big difference between drinking very quickly versus drinking and holding or drinking and swishing in the mouth, which significantly increases the ability of these compounds to induce acid wear on dental enamel through their action on hydroxyapatite crystals, ultimately weakening the enamel. This process may be attenuated by the buffering capacity and mineral content of saliva.^41^

Epidemiological studies involving adolescents have revealed prevalence rates of dental erosion exceeding 30%, with higher susceptibility in males and an increasing trend with age.^29,43^ Although erosion mainly affects enamel, preventive strategies based on solid evidence are essential. Given the increased frequency of soft drink consumption among adolescents, previous systematic reviews have revealed a positive association between the consumption of carbonated beverages and the development of dental erosion.^7,27^ However, the evidence remains inconsistent because some studies have failed to find statistically significant associations. For instance, in an analysis of NHANES 2003–2004 data, Samman et al^44^ reported no clear link between carbonated beverage consumption and erosive tooth. These discrepancies underscore the need for more robust evidence to clarify the relationship between carbonated beverages and dental erosion.

Therefore, the objective of this study is to present the global prevalence and a synthesis of current evidence on the relationship between dental erosion and carbonated drink consumption among adolescents through a systematic review and meta-analysis. The results of this study can be used in the design of preventive programmes that can reduce the impact of dental erosion in this vulnerable age group.

## MATERIALS AND METHODS

The protocol for this systematic review was designed by all the authors. This study was registered at the National Institute for Health Research, PROSPERO, International Prospective Register of Systematic Review (ID: CRD42023398708) and was designed in accordance with the preferred reporting items for systematic review and meta-analysis (PRISMA) guidelines.^38^

### Search Strategy and Study Selection

All relevant literature published between 2000 and 17 December 2023 was systematically extracted from five international databases, including PubMed, Web of Science, Scopus, the Latin American and Caribbean Health Sciences Literature database (LILACS), and SciELO. Medical subject headings (MeSH) and free terms were combined according to the syntax rules for each database. Terms related to dental erosion and risk factors were searched. Our search strategy included the following terms: ‘tooth erosion’, ‘dental erosion’, ‘adolescents’, and ‘carbonated drinks’, with the Boolean indicators AND, OR, with the research question: What is the evidence on carbonated beverages as a risk factor for dental erosion in adolescents? Moreover, we manually screened potentially relevant publications from the references of our retrieved studies. This process was performed independently by two participants (MLJL and FG).

### Participants and Eligibility Criteria

The study’s inclusion criteria followed a PEO framework: used to identify dental erosion and non-erosion (outcome) and intake of carbonated beverages (exposure) in adolescents (population). The odds ratios (ORs) with 95% confidence intervals (CIs) were considered or computed if enough relevant data were available. We included studies that were properly registered and approved by their relevant ethics committees; case–control, cross-sectional, and cohort studies were utilised. Only studies in English and Spanish were included.

Studies were excluded if they were case reports, reviews, summaries of discussions, or in vitro studies; if they contained insufficient data for analysis, or if the patients were stratified based on the degree of severity.

In this study, the diagnostic criterion was limited to the detection of the presence or absence of erosive dental lesions, without considering the extent or severity of tissue loss. The use of various clinical indices in adolescent populations is justified by their ability to capture early and clinically meaningful signs of dental erosion. Table 1 presents the primary diagnostic features of the indices applied in adolescent cohorts, all of which consider the identification of initial erosive changes as a central criterion; all the indices detect early lesions. For the purposes of this analysis, carbonated beverages were operationally defined as sugar-containing drinks characterised by effervescence.

**Table 1:** Criteria for dental erosion indices

Index	Author/year	Surfaces evaluated	Scoring scale	Detect other types of wear	Scope of use
BEWE^30^	Lussi et al, 2008	Dental sextants (most affected tooth per sextant)	0 to 3	No	Clinical and epidemiological
Johansson and Carlsson^23^	Johansson et al, 2003	Buccal and occlusal surfaces	0 to 3	No	Epidemiological (adolescents)
O’Sullivan^37^	O’Sullivan, 2000	Upper incisors and permanent molars	0 to 4	No	Epidemiological (children/adolescents)
Lussi^28^	Lussi, 2000	Buccal, palatal, and occlusal surfaces	0 to 3	No	Clinical and experimental
Smith and Knight (TWI)^49^	Smith and Knight, 1984	All tooth surfaces	0 to 4	Yes	Clinical and academic
Eccles^12^	Eccles, 1979	Incisors and molars	0 to 3	No	Clinical


To evaluate publication bias, we examined the symmetry of the funnel plot and evaluated the intercept of publication bias quantitatively using Egger’s regression test. Additionally, only studies that evaluated the association between dental erosion and the consumption of this kind of drink more than once daily or more than seven times weekly were included in the meta-analysis. These exposure thresholds were regarded as equivalent and ensured consistent classification of high-frequency intake across the selected studies.

### Data Extraction and Quality Assessment

The literature screening, data extraction, and literature quality evaluation were conducted separately by two analysts (MLJL and FG). Any differences were resolved through mutual discussion or consultation with a third analyst (LC).

A data extraction spreadsheet was designed, and the following information was extracted: first author’s surname, year of publication, country, participant characteristics, and statistical summaries related to factor risk (carbonated drinks).

### Risk of Bias

The quality of each included study was independently assessed by the two analysts to evaluate the possible risk of bias, and the Newcastle–Ottawa scale (NOS), as it provides a comprehensive framework for observational studies that focuses on three key domains: selection of study groups, comparability of groups, and outcome assessment to obtain a final score.^35^ The NOS is a star rating system that assigns a maximum of nine stars across three categories. Studies scoring three or four stars in selection, one or two stars in comparability, and two or three stars in outcome/exposure were considered of good quality. Studies with two stars in selection, one or two stars in comparability, and two or three stars in outcome/exposure were classified as fair quality. When the studies scored no stars or one star in selection, no stars in comparability, and no stars or one star in outcome/exposure, they were considered of poor quality. In accordance with this method, a study is considered to have a low risk of bias (8 or 9 stars), a fair risk (5–7 stars), or a high risk of bias (< 5 stars). In this review, 24 studies with scores ≥ 5 were included for analysis.

### Statistical Analysis and Meta-Analyses

A standardised data extraction form was used for qualitative analyses to record the study characteristics (author and publication year), study design, and main findings. The meta-analysis of prevalence was conducted using MedCalc software version 23.0.2 (MedCalc Software), applying the Freeman–Tukey’s arcsine square-root transformation and the DerSimonian and Laird random-effects model. Publication bias was assessed through Egger’s test.

To evaluate the association between dental erosion and carbonated drink consumption, OR and 95% confidence interval (95%CI) were calculated. The meta-analysis of ORs was performed using Review Manager (RevMan) software version 5.4.1 (Cochrane Collaboration),^42^ using the Mantel–Haenszel (M–H) method with a DerSimonian and Laird random-effects model. The I^2^ statistic was used to assess heterogeneity among studies, with values of ≤ 25%, 25–50%, 50–75%, and > 75% indicating no, low, moderate, and significant heterogeneity, respectively.

For both meta-analyses, forest plots were generated, and a *P* value < 0.05 was considered statistically significant.

## RESULTS

### Selection, Characteristics, and Quality of the Studies

**Fig 1:**
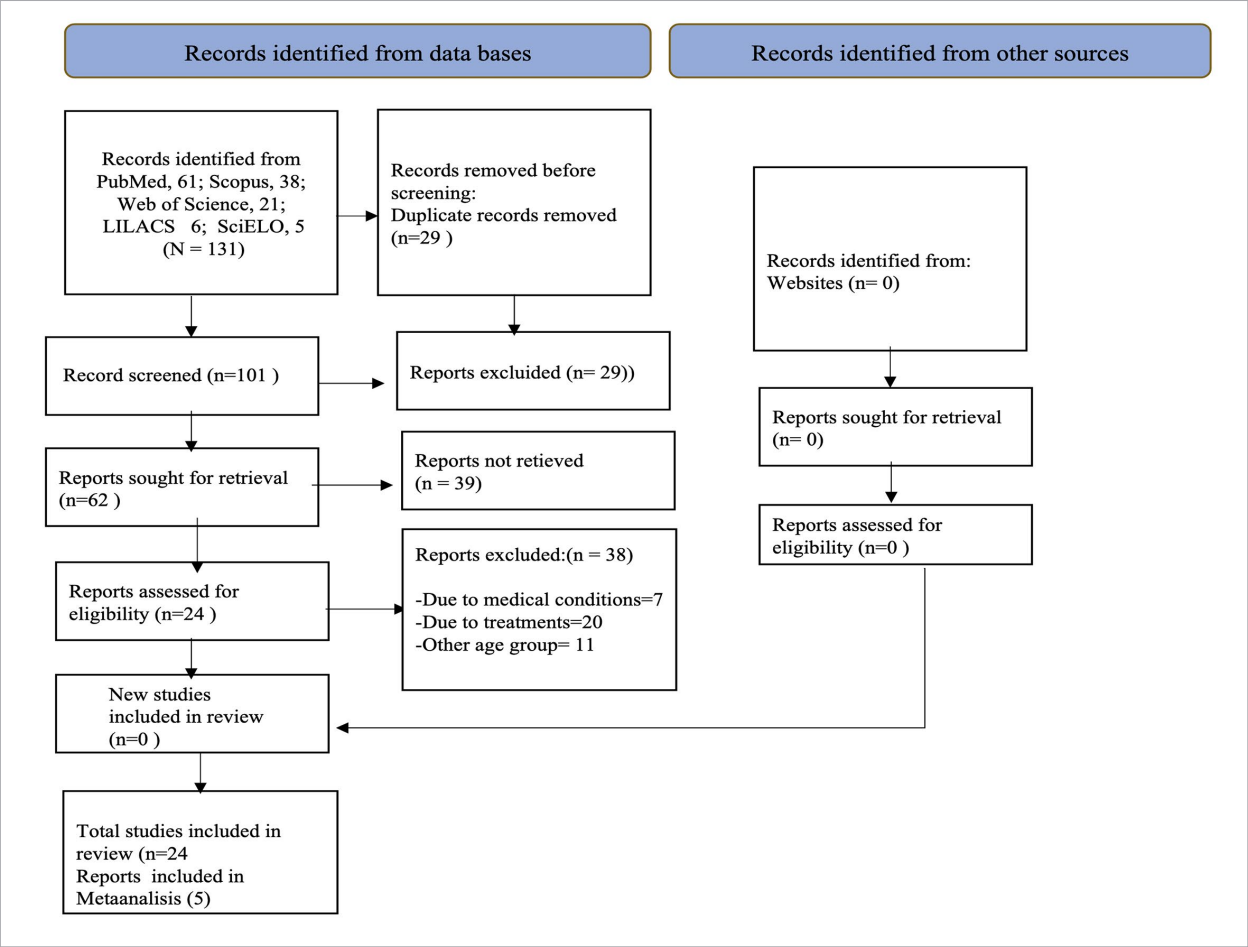
PRISMA flow diagram.

A total of 131 articles were identified; after removing duplicates, 101 records remained. Titles and abstracts were screened for the eligibility criteria, resulting in the selection of 24 full-text articles for qualitative analysis. Among the included articles, 21 were cross-sectional studies, 2 were case–control studies, and 1 was a cohort study. For the prevalence meta-analysis, cross-sectional studies were used. To assess the association between dental erosion and carbonated beverages, only five articles were considered for the quantitative analysis of risk factors because they could be combined (Fig 1).

### Qualitative Analysis

Table 2 presents the main findings from the included studies, which involved 21,541 adolescent participants. The reported prevalence of dental erosion ranged from 1.4% to 80%. The average participant age was 15.0 ± 2.7 years.

**Table 2:** Summary of the studies on dental erosion and soft drink consumption

Author, year, country	Study design	Sample characteristics and index used	Findings
González-Aragón et al^17^ (2022), Mexico	Case‒control study	n = 291, age 13–14 yearsIndex: BEWE	The case and control groups were conformed each one by n = 97 subjects. The BEWE score for cases with marked defects was 4.77 ± 0.93; the score for mild cases was 2.01 ± 0.54. Weekly sweetened carbonated drink intake: 3.22 ± 4.18 (cases) vs 1.98 ± 1.96 (controls). OR 1.16, 95%CI: 1.03–1.31, *P* = 0.012. Weekly milk intake was a protective erosion factor. *P *= 0.043.
Jász et al^22^ (2022), Hungary	Cross-sectional	n=579, age 12 yearsIndex: BEWE	Prevalence: 21.2%.Higher scores were observed in urban areas.A correlation was observed between erosion and daily cola/carbonated beverage consumption (*P* = 0.034), which was also associated with maternal education level.
Korkmaz et al^25^ (2020), Turkey	Cross-sectional	n = 473, age 11–14 yearsIndex: O’Sullivan	Prevalence: 21%.Cola beverage: OR 3.6 (95%CI: 2.04–6.51), soda: OR 2.14 (95%CI: 1.12–4.08), energy drinks: OR 10.1 (95%CI: 1.34–75.25).Higher erosion in students who sipped slowly, retained drinks in mouth, or drank before bedtime (*P* < 0.05).
Simangwa et al^48^ (2019), Tanzania and Uganda	Cross-sectional	n = 906, age 12-17 yearsIndex: Johansson y Carlsson	Prevalence: 30%.Association with carbonated drinks: OR 1.6 (95%CI: 1.3–2.1). Fluorosis and caries are also reported.
Marro et al^31^ (2018), Belgium	Cross-sectional	n = 613, age 13-17 yearsIndex: BEWE	Prevalence: 48.6%.Association with carbonated beverages > 1/day: OR 2.13 (95%CI: 1.38–3.28), *P* = 0.001.School environment may influence dietary habits and the prevalence of erosion.
Skalsky et al^47^ (2018), Sweden	Nested Cross-sectional	n = 1335, age 15-17 yearsIndex: SEPRS Simplified erosion partial recording system	Prevalence: 31.4%.Higher soft drink intake several times per week was associated with erosion (*P* < 0.001).OR 1.65 (95%CI: 1.25–2.15), *P* = 0.001.Also linked to lifestyle factors such as consumption of juices or sports drinks after exercise.
Harlukowicz et al^18^ (2017), Poland	Cross-sectional	n = 240, age 12-18 yearsIndices: Lussi, O’Sullivan, BEWE	Prevalence: 16.25%.BEWE score: 2.23 ± 1.42.Spearman correlation reported with erosion in oclusal surfaces *P *< 0.05.
Provatenou et al^40^ (2016), Greece	Cross-sectional	n = 263, age 14 yearsIndex: BEWE	Prevalence: 21%.Lesions were mostly in enamel.Carbonated drink consumption was associated with erosion.OR 2.11 (95%CI: 1.0–4.30), *P* < 0.05.
Shahbaz et al^46^ (2016), Pakistan	Cross-sectional	n = 385, age 12–14 yearsIndex: BEWE	Prevalence: 46%.Consumption > 8 times/week associated with erosion.OR 3.87 (95%CI: 1.43–10.49), *P* < 0.001.Brushing after drinks resulted in increased erosion.
González et al^16^ (2016), Mexico	Cross-sectional	n = 417, age 14–19 yearsIndex: Lussi	Prevalence: 31.7%.Associated with frequent sweetened soda intake (≥4 times/week): OR 1.80 (95%CI: 1.03–3.07), *P* = 0.03.Also associated with xerostomia and posterior molar erosion.
Kirthiga et al^24^ (2015), India	Cross-sectional	n = 2000, age 11–16 yearsIndex: O’Sullivan	Prevalence: 1.4%.No association with carbonated drink intake.Considered personal demographic data and acidic food/drink habits.
Al-Hadi et al^2^ (2013), Jordan	Cross-sectional	n = 3812, age 12 yearsIndex: Smith and Knight	Prevalence: 32.2%.Association with carbonated drinks. < 0.01 and drinking before bedtime, OR 7.8 (95%CI: 3.94–15.42), retaining drinks in mouth, sports beverages, swimming, systemic diseases, steroid inhalation, regurgitation, and antacid use.
Chrysanthakopoulos et al^9^ (2012), Greece	Cross-sectional	n = 770, age 13–16 yearsIndex: BEWE	Prevalence: 33.8%.Associated with carbonated drink intake, OR 3.99 (95%CI: 1.37–11.59, *P* = 0.011).Some adolescents retained drinks in their mouth before swallowing; night-time acidic beverage consumption was also associated.
El Aidi et al^13^ (2011), Netherlands	Cohort	n = 572, age 11 yearsIndex: Lussi	Prevalence: 42%.Associated with carbonated drinks, OR 1.04 (95%CI: 1.01–1.07, *P* = 0.016).Greater prevalence in molars vs incisors.Also linked to bruxism and mixed alcoholic beverages.
Kumar et al^26^ (2011), India	Cross-sectional	n = 605, age 11–14 yearsIndex: O’Sullivan	Prevalence: 8.9%.Associated with lemon drink intake several times/day, OR 13.4 (95%CI: 1.5–116.7, *P* < 0.001) and carbonated drinks, OR 2.8 (95%CI: 1.32–5.92, *P* = 0.007).
Bardolia et al^6^ (2010), UK	Cross-sectional	n = 629, age 13 yearsModified Partial Index	Prevalence: 30.62%.Carbonated drink intake > 1/day associated with erosion, OR 1.6 (95%CI% 1.1–2.3).More frequent in males.
Okunseri et al^36^ (2010), USA	Cross-sectional	n = 1314, age 13–19 yearsIndex: Smith and Knight	Prevalence: 45%. No individual association was found, but the adjusted model showed associations with male sex, age, and higher intake of juices and carbonated drinks, OR 1.24 (95%CI: 1.08–1.43, *P* = 0.03).
Hasselkvist et al^19^ (2010), Sweden	Cross-sectional	n = 474 (227 aged 13–14; 247 aged 18–19)Index: Smith and Knight (TWI)	Prevalence: 34.4%.Erosion severity was greater in 18–19-year-old boys (*P* < 0.05).Carbonated drink intake correlated with erosion severity.
Wang et al^53^ (2010), China	Cross-sectional	n = 1499, age 12–13 yearsEccles and O’Sullivan Index	Prevalence: 27.3%.Higher erosion in adolescents drinking sodas > 1/week.Most affected: maxillary central incisors, incisal or occlusal surfaces.OR 1.29 (95%CI: 1.028–1.64), *P* = 0.02.
Waterhouse et al^54^ (2008), Brazil	Cross-sectional	n = 458, age 13 yearsIndex: Smith and Night	Prevalence: 34.1%.Carbonated drink consumption associated with erosion.OR 1.71 (95%CI: 1.12–2.62), *P* = 0.014.
Milosevic et al^34^ (2004), UK	Cross-sectional	n = 2385, age 14 yearsTooth Wear Index	Prevalence: 27.04%.OR 1.32 (95%CI: 1.08–1.62), *P* < 0.0001.Also examined relationship with other foods and frequency of consumption of various beverages.
Árnadóttir et al^5^ (2003), Iceland	Case-control	n = 278, age 15 yearsIndex: BEWE	The case and control groups were conformed by n = 60 subjects each one.High-risk threshold: > 800 ml/day.No association with carbonated drinks was found (OR 1.7, 95%CI: 0.5–5.0, *P* > 0.05).Lesions were classified by location.
Al-Majed et al^3^ (2002), Saudi Arabia	Cross-sectional	n = 354 (age 5–7), n = 862 (age 12–14)Index: Smith and Knight (TWI)	Prevalence: 95%.Risk associated with consuming carbonated drinks before bedtime.In 5–6-year-olds, erosion was associated with sugary drink intake.In 12–14-year-olds, erosion was observed in permanent teeth due to consumption of various sugary drinks (*P* = 0.02).
Al-Dlaigan et al^1^ (2001), UK	Cross-sectional	n = 418, age 14 yearsIndex: BEWE	Prevalence: 80%.Associated with intake of beer, sports drinks, apple, strawberry, vinegar, salad dressing.Spearman correlation. Erosive lesions were associated with cola and other carbonated beverage consumption (*P* < 0.001).


There was variability in the diagnostic index for erosion; eight studies used the BEWE index, three used Lussi’s index, six used the Smith and Knight index (TWI), four used O’Sullivan’s index, one used the Carlsson index, and two used the simplified erosion teeth partial recording system.

An analysis of the included studies revealed that 19 (74%) reported a statistically significant association between carbonated beverages and dental erosion. In the remaining 26%, the association was either not statistically significant or was the result of exposure to a combination of various acidic beverages.

### Quantitative Analysis

**Fig 2:**
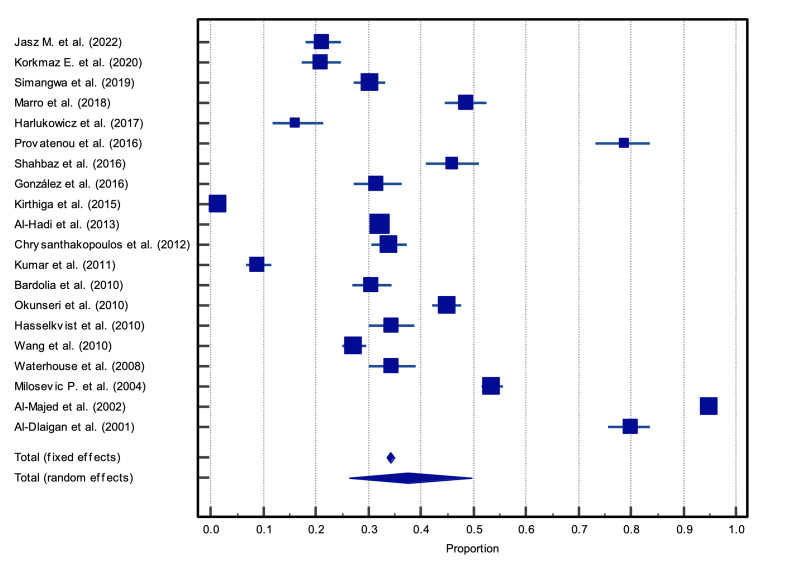
Forest plot of the prevalence of dental erosion, 37.6% (95%CI: 26.6 to 49.7%).

The random-effects meta-analysis of prevalence revealed that 37.6% (95%CI 26.3, 49.7%) of the participants in the included studies had dental erosion (Fig 2).

**Fig 3:**
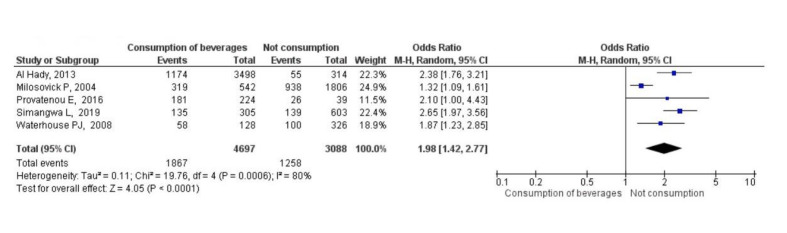
Association between carbonated beverage consumption more than once a day and dental erosion among adolescents.

In addition, the pooled results indicated that adolescents who consumed carbonated beverages had a 98% grater of experiencing dental erosion (OR = 1.98, 95% CI: 1.42–2.77; 7785 participants; *P* = 0.0001). However, the observed heterogeneity (I^2^ = 80%) reflects differences among the included studies (Fig 3). Because the consumption of carbonated beverages by gender is not reported in primary studies, it was not possible to carry out a stratified analysis.

### Risk of Bias

The quality of 20 studies was good; four studies were classified as fair quality and bias, one of which shows a total score of five stars. The comparability of participants was less fulfilled by 11 studies, being the category of lowest compliance, increasing the risk of bias (Table 3).

**Table 3:** Quality of the included studies

Author	Year	Country	Selection	Comparability	Outcome	Total	Quality		Bias
González-Aragón et al^17^	2022	Mexico	★★★	★	★★★	7	Good		Low
Jasz et al^22^	2022	Hungary	★★★	★	★★★	7	Good		Low
Korkmaz et al^25^	2020	Turkey	★★★	★★	★★	7	Good		Low
Simangwa et al^48^	2019	Tanzania & Uganda	★★★	★★	★★	7	Good		Low
Marro et al^31^	2018	Belgium	★★★	★★	★★	7	Good		Low
Skalsky et al^47^	2018	Sweden	★★	★★	★★	6	Fair		Fair
Harlukowicz et al^18^	2017	Poland	★★	★★	★★	6	Fair		Fair
Provatenou et al^40^	2016	Greece	★★★	★★	★★	7	Good		Low
Shahbaz et al^46^	2016	Pakistan	★★	★	★★	5	Fair		Fair
González et al^16^	2016	Mexico	★★★	★	★★★	7	Good		Low
Kirthiga et al^24^	2015	India	★★★	★★	★	6	Good		Fair
Al-Hadi et al^2^	2013	Jordan	★★★	★	★★★	7	Good		Low
Chrysanthakopoulos et al^9^	2012	Greece	★★★	★	★★	6	Good		Fair
El Aidi et al^13^	2011	Netherlands	★★★	★	★★★	7	Good		Low
Kumar et al^26^	2011	India	★★	★★	★★	6	Fair		Fair
Bardolia et al^6^	2010	United Kingdom	★★★	★★	★	6	Good		Fair
Okunseri et al^36^	2010	USA (Wisconsin)	★★★	★★	★	6	Good		Fair
Hasselkvist et al^19^	2010	Sweden	★★★	★★	★★	7	Good		Low
Wang et al^53^	2010	China	★★★	★★	★★	7	Good		Low
Waterhouse et al^54^	2008	Brazil	★★★★	★	★★	7	Good		Low
Milosevic et al^34^	2004	United Kingdom	★★★	★★	★	6	Good		Fair
Árnadóttir et al^5^	2003	Iceland	★★★	★	★★	6	Good		Fair
Al-Majed et al^3^	2002	Saudi Arabia	★★★★	★	★★	7	Good	Low	
Al-Dlaigan et al^1^	2001	United Kingdom	★★★	★	★★	6	Good	Fair	
★ = 1 point according to the Newcastle–Ottawa Scale (NOS).									

**Fig 4:**
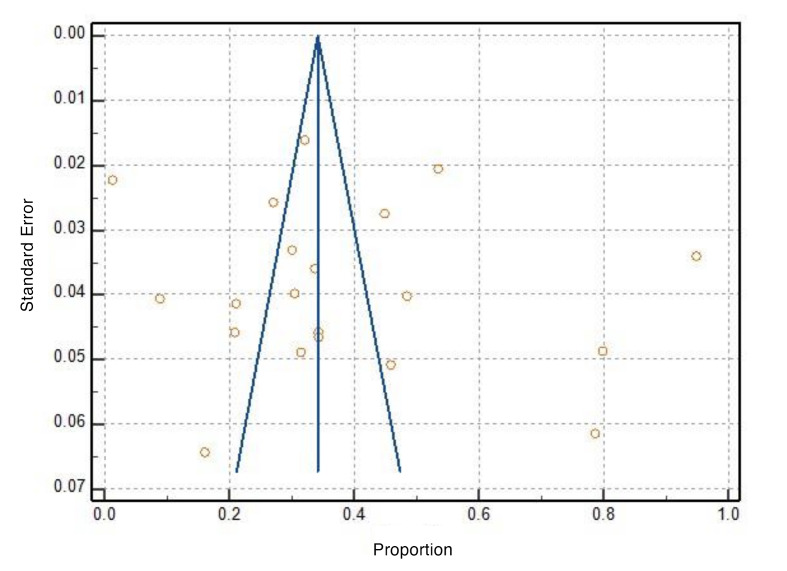
Funnel plot to evaluate the publication bias. Result of Egger test 7.38 (95%CI: ‒14.05 to 28.81) *P* = 0.479.

Publication bias was assessed using funnel plot (Fig 4) and Egger’s test: 7.38 (95%CI: –14.05 to 28.81, *P* = 0.479). The funnel plot and the intercept value suggest asymmetry. However, the statistical analysis does not provide strong evidence of publication bias.

## DISCUSSION

In this systematic review, the prevalence and the association between the consumption of carbonated beverages and dental erosion were analysed. The findings indicate that almost 4 out of 10 adolescents present signs of erosive tooth wear. However, prevalence rates vary across countries because of cultural differences and the age range of study participants; older adolescents tend to have higher prevalence rates, likely because of prolonged exposure, a greater number of affected teeth, and more severe lesions.^16,46^ Previous studies have also indicated that male adolescents are at higher risk, potentially because they had a higher level of soft drinks and cola ingestion than girls, and a greater frequency of acidic drink consumption linked to sports and recreational activities.^19,31,36^ In the present analysis, this relationship resulted controversial while some studies point it out,^19,24,34,35,36,40^ others do not^20,23,26,27,31,33^ and even point out that there is greater erosion in adolescent women^37^; although we could not make the analysis by gender because primary studies do not present stratified results.

The current findings align with those reported by Li^27^ and Marschner,^32^ who showed that erosive lesions were not only associated with carbonated beverages but also with digestive disorders, regurgitation, vitamin C intake, and the consumption of acidic foods such as sauces. The erosive potential of carbonated drinks is primarily attributed to their low pH (typically between 2.3 and 3.5), which is far below the critical threshold of 5.5 for enamel demineralisation. This acidity is exacerbated by the presence of phosphoric, citric, and carbonic acids, which promote the dissolution of hydroxyapatite, leading to progressive enamel softening and increased surface roughness. Additionally, these beverages often exhibit high titratable acidity, which prolongs the time required for the salivary pH to return to neutral, further increasing the risk of mineral loss.^10,21^

The difference between the previous analysis of Salas et al^43^ and Li et al^27^ is that the present review focused specifically on carbonated beverages, given their high consumption among adolescents. International data have reported that 16% of adolescents drink carbonated beverages daily, with higher intake associated with lower socioeconomic status.^33^ Another study suggests that more than half of adolescents aged 12 to 15 in low- and middle-income countries consume these beverages at least once daily.^21^ A global analysis further supported this finding, indicating a 43% daily consumption rate among youth aged 12 to 17. Other erosive beverages commonly preferred by adolescents include energy drinks and citrus-based juices.^32,53^ Therefore, this analysis highlights the effects on dental tissues of these dietary patterns. Moreover, soft drink intake has also been linked to an increased risk of several medical conditions.^51^

Several behavioural and environmental factors modulate the erosive impact of these beverages. Marro et al described a ‘school environment halo effect’ that influences dietary habits. The erosive risk increases significantly when acidic drinks are consumed at night or sipped slowly and retained in the mouth.^31^ These habits are common in adolescents, especially post-exercise, when salivary flow is reduced, and buffering capacity is compromised. Salivary factors, along with protective dietary elements such as dairy products, play a key role in modulating erosion. Saliva contributes to pH regulation and enamel remineralisation.^41^ Gonzalez et al reported a correlation between dental erosion and xerostomia.^16^

The pattern and severity of erosion are influenced by the frequency and volume of beverage intake, as well as individual protective factors like consumption of milk-based drinks.^29^ The manner of drinking (savouring vs swallowing) also affects the degree of damage. Bardolia et al emphasised that frequent and prolonged exposure to acidic beverages causes repeated episodes of low pH, contributing to progressive enamel wear.^6^ Chan reported that the most consistent findings indicate the erosive potential of carbonated beverages and the consumption of acidic drinks at bedtime.^7^

Clinically, dental erosion leads to dentin hypersensitivity, masticatory discomfort, and increased susceptibility to fractures, potentially resulting in early tooth loss. Erosive lesions caused by carbonated beverages are most frequently observed on the occlusal surfaces of posterior teeth and the palatal surfaces of maxillary and mandibular central incisors. These findings highlight the need for targeted preventive strategies emphasising the reduction of acidic beverage consumption in schools and youth settings. Health professionals should promote awareness of the risks posed by acidic drinks and advocate for early detection and the consumption of water and protective foods such as milk, cheese, and yoghurt, which provide calcium and phosphate. Preventive interventions such as topical fluorides, CPP-ACP complexes, and calcium-fortified beverages are also recommended.^8,15,20^

Samman’s cluster analysis underscored the complexity of beverage consumption behaviours, demonstrating that individuals do not consume beverages in isolation throughout their daily routines. Beverages should therefore be considered collectively, as they may exert complementary or antagonistic effects. In the case of diet drinks, several potential health risks have been reported, including systemic conditions such as dementia, diabetes, vascular and metabolic diseases, and stroke.^44^

Variability in the operational definitions of exposure to carbonated beverages across studies was a major contributor to methodological heterogeneity. Whereas some investigations estimated risk based on consumption frequency, others used measures of daily intake volume. This inconsistency in exposure assessment limited comparability across studies and constrained quantitative data synthesis, resulting in only 25% of eligible studies meeting the homogeneity criteria for inclusion in the meta-analysis of the association. In addition, considerable heterogeneity was observed in the meta-analysis (I^2^ = 80%), indicating substantial between-study variability beyond that expected by chance. Accordingly, the pooled effect estimate should be interpreted with caution, as it may be influenced by differences in study design, population characteristics, exposure definitions, and outcome assessment methods.

This study has several limitations, such as the fact that most of the included studies were cross-sectional in design and relied on self-report questionnaires. This introduces potential recall bias and may lead to the overestimation of associations. Sample size was another methodological issue; small samples reduce statistical power, whereas very large samples can yield statistically significant but clinically irrelevant results. Additionally, compared with cohort studies, cross-sectional studies are more likely to reveal significant associations because of their lack of temporal sequencing, potentially leading to overestimation.^4,39,45^ The asymmetry observed in the funnel plot should be interpreted with caution, as it does not constitute definitive evidence of publication bias. Although publication bias is a recognised explanation, such asymmetry may also stem from alternative sources, including methodological variations.^11^

Dental erosion is clearly a multifactorial disease, and further investigation into the relative contribution of associated factors, such as taste preferences linked to a higher intake of carbonated beverages. Nevertheless, the clear correlations observed in the present study provide evidence supporting dietary interventions as a key component in the development of preventive strategies for dental erosion.

Given the limited number of studies included in the meta-analysis, the results should be interpreted cautiously. Future research should employ longitudinal designs, standardised diagnostic criteria, and validated questionnaires to provide stronger evidence regarding the association between carbonated beverage consumption and dental erosion.

## CONCLUSION

Erosive tooth wear affects 37% of adolescents and represents a growing public health concern. Frequent consumption of carbonated beverages, particularly more than once daily, has been consistently identified as a risk factor. Accordingly, adolescent health promotion strategies should prioritise reducing the intake of these kinds of drinks.

### Acknowledgements

#### Support

This research was supported by Dirección General de Asuntos del Personal Académico, Universidad Nacional Autónoma de México (DGAPA-UNAM) (PAPIME PE 201525).

#### Conflict of interest

The authors declare no conflict of interest.

#### Data availability

The data presented in this study are available on request from the corresponding author.

## REFERENCES
